# Exploration of molecular genetic etiology for Korean cochlear implantees with severe to profound hearing loss and its implication

**DOI:** 10.1186/s13023-014-0167-8

**Published:** 2014-11-06

**Authors:** Joo Hyun Park, Nayoung K D Kim, Ah Reum Kim, Jihye Rhee, Seung Ha Oh, Ja-Won Koo, Jae-Yong Nam, Woong-Yang Park, Byung Yoon Choi

**Affiliations:** Department of Otorhinolaryngology-Head and Neck Surgery, Dongguk University Ilsan Hospital, Goyang, Korea; Samsung Genome Institute, Samsung Medical Center, Seoul, Korea; Department of Otorhinolaryngology-Head and Neck Surgery, Seoul National University Hospital, Seoul, Korea; Sensory Organ Research Institute, Seoul National University Medical Research Center, Seoul, Korea; Department of Otorhinolaryngology-Head and Neck Surgery, Seoul National University Bundang Hospital, College of Medicine, Seoul National University, Bundang-gu Seongnam-si, Korea; Samsung Advanced Institute for Health Sciences and Technology, Seoul, Korea; Department of Molecular Cell Biology, School of Medicine, Sungkyunkwan University, Seoul, Korea

**Keywords:** Molecular genetic test, Cochlear implantation, Deafness, Targeted resequencing

## Abstract

**Background:**

Severe to profound sensorineural hearing loss (SNHL) requires cochlear implantation (CI) for auditory rehabilitation. Etiologic diagnoses can contribute to candidacy selection and decision-making regarding the timing of successful CI. However, few studies have been performed to address the etiologic spectrum of severe SNHL in the population where there is no consanguineous marriage and the majority of SNHL cases are sporadic in small sized families. The authors sought to comprehensively understand the etiologies of Korean cochlear implantees by incorporating the targeted resequencing of 204 candidate deafness genes (TRS-204) and a phenotype-driven candidate gene approach.

**Methods:**

Ninety-three that consented to molecular genetic testing and underwent at least one molecular genetic test were included. Patients with a characteristic Phenotypic marker were subject to Sanger sequencing to detect variants in corresponding candidate genes. The rest of patients without any prominent phenotype were tested on *GJB2.* Next, TRS-204 was applied in *GJB2*-negative cases without any phenotypic marker. In addition, the sibling recurrence-risk of SNHL among families with non-diagnostic genotypes after TRS-204 was performed to gain insight of etiologies in non-diagnostic cases.

**Results:**

Overall, we could find causative variants in 51 (54.8%) of the 93 cochlear implantees. Thirty (32.3%) probands could be diagnosed by direct Sanger sequencing of candidate genes selected by their phenotypes. *GJB2* sequencing added 10 subjects to the group with a diagnostic genotype. TRS-204 could detect a causative variant from additional 11 cases (11.8%). We could not detect any pathogenic deletion or duplication on 204 target genes. The sibling recurrence-risk of SNHL among 42 genetically undiagnosed families with 0.03 (1/38) was significantly lower than among genetically diagnosed recessive families with 0.19 (7/37).

**Conclusion:**

Despite that the majority of severe or more degree of SNHL occurs sporadically in Koreans, at least 54.8% of such cases that were willing to join the genetic study in the Korean population are monogenic Mendelian disorders with convincing causative variants. This study also indicates that a substantial portion of unsolved cases after applying our current protocol are predicted to have non-genetic or complex etiology rather than a Mendelian genetic disorder involving new genes beyond the 204 target genes.

**Electronic supplementary material:**

The online version of this article (doi:10.1186/s13023-014-0167-8) contains supplementary material, which is available to authorized users.

## Background

Given advances in cochlear implantation (CI) and the extension of indications for the procedure, more attention has been focused on the etiology of deafness and its potential impact on CI outcomes [1–3]. The etiology of sensorineural deafness manifests extreme heterogeneity. A rigorous clinical evaluation involving audiological tests, imaging, and laboratory tests, including molecular genetic tests, is needed to reach precise etiologic diagnoses [4,5]. Understanding genetic etiology can provide valuable clues of prognosis (i.e., whether losses will worsen), optimal intervention (e.g., hearing aids, CI, sign language), and the risk of hearing loss recurrence in future children and other family members [6–9]. For example, in hereditary deafness with mutations of specific genes, such as, *GJB2*, *SLC26A4*, *OTOF*, *COCH*, and *MYH9*, mitochondrial mutations, CI is expected to provide successful results [6].

Genetic hearing loss has been estimated to be responsible for more than half of congenital bilateral profound deafness cases [10,11], however no definitive means of confirming this estimate so far. Furthermore, because of the extreme heterogeneities of causative genes, studies of molecular etiology have focused mainly upon deafness genes, such as, *GJB2*, *SLC26A4*, or *OTOF*, which make larger contributions to deafness in local populations. Several phenotypic markers that previously turned out to be highly prone to genetic alterations, such as, enlarged vestibular aqueduct or incomplete partition type III inner ear anomaly, have also facilitated the clarifications of genetic etiologies. Nevertheless, these approaches are able to explain only a subset of CI candidates, which means that a substantial proportion remain elusive in terms of etiology.

Recently, the advent of next generation sequencing (NGS) technologies has markedly influenced our strategy regarding the genetic diagnosis of deafness. In particular, the targeted resequencing (TRS) of known deafness genes (panel sequencing) based upon NGS allows a greater number of deafness samples to be examined with the advantages of significant cost and turnaround time saving [12]. Novel genes causing non-syndromic hearing loss [13–15] and syndromic hearing loss [16,17] have been successfully discovered by this targeted NGS approach, and several groups, including ours, have reported on the efficacy of this approach for the high-throughput screening of mainly multiplex autosomal dominant families [18-20]. However, its usefulness for the mass-screening of sporadic or potentially autosomal recessive congenital severe to profound hearing loss (a main indication for CI) has not been extensively studied. Hearing loss in 32% (69/216) of Japanese cochlear implantees was recently explained genetically using a molecular genetic test protocol incorporating their Invader assay and TRS [3] but no clue was unearthed regarding the remaining unsolved cases.

In the present study, we refined our phenotype-driven candidate gene approach and combined it with a targeted NGS approach employing the large deafness panel in a hierarchical manner to determine the genetic etiologic spectrum of cochlear implantees. The targeted NGS data were also reviewed to uncover, if any, the presence of copy number variation in the targeted genes. In addition, we could propose a clue to uncover the etiology of each case that still remained unanswered after the targeted NGS approach by calculation of the sibling recurrence risk of hearing loss.

## Methods

### Study participants

We initially recruited 236 unrelated subjects who had undergone cochlear implantation between May 2010 and August 2012 at two tertiary referral hospitals (Seoul National University Bundang Hospital and Seoul National University Hospital). The following exclusion criteria were applied; a history of non-genetic risk factors for hearing loss, such as, stay in a neonatal intensive care unit stay of >48 hours, prematurity, hypoxia, hyperbilirubinemia, an *in utero* infection (e.g., symptomatic and confirmed asymptomatic cytomegalovirus (CMV), herpes, rubella, toxoplasmosis), a postnatal infection (e.g., meningitis), exposure to ototoxic medications, head trauma, and recurrent otitis media, they were excluded. Since a substantial portion of our CI candidates were referred from hospitals all around in this country, symptomatic CMV patients with hearing loss have already been diagnosed before visit to our clinics. However, asymptomatic CMV cases would have been missed, since we were not able to access blood or saliva samples of the patients taken within 3–4 weeks after birth. Asymptomatic CMV infection was diagnosed only in a subset of cases with preserved dried blood spot or dried umbilical cords taken at birth. Under these criteria, 26 of 236 subjects were excluded. In addition, 107 subjects refused to join the molecular genetic study due to reluctance to disclose that hearing loss is genetic or to make additional visit to our genetic hearing loss clinics especially when patients live far away from our hospital. Resultantly, among the 236 subjects, 93 subjects who consented to the molecular genetic test and went through at least one molecular genetic test were finally included. There was no difference in the proportion of multiplex hearing loss families between the recruited 93 families and the other 107 families. The majority of families were singleton cases in both groups.

### Ethics statement

This study was approved by the Institutional Review Boards (IRBs) of Seoul National University Bundang Hospital (IRB-B-1007-105-402) and Seoul National University Hospital (IRBY-H-0905-041-281). Written informed consent was obtained from all participants. For children, written informed consent was provided by parents or guardians.

### DNA preparation and phenotype-driven candidate gene approach

**Figure 1:**
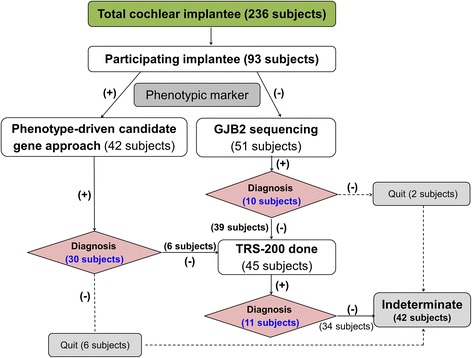
**Flow diagram of our hierarchical molecular genetic test in cochlear implantees.** This flow diagram represents a protocol for genetic testing in patients with severe to profound hearing loss.

**Figure 2:**
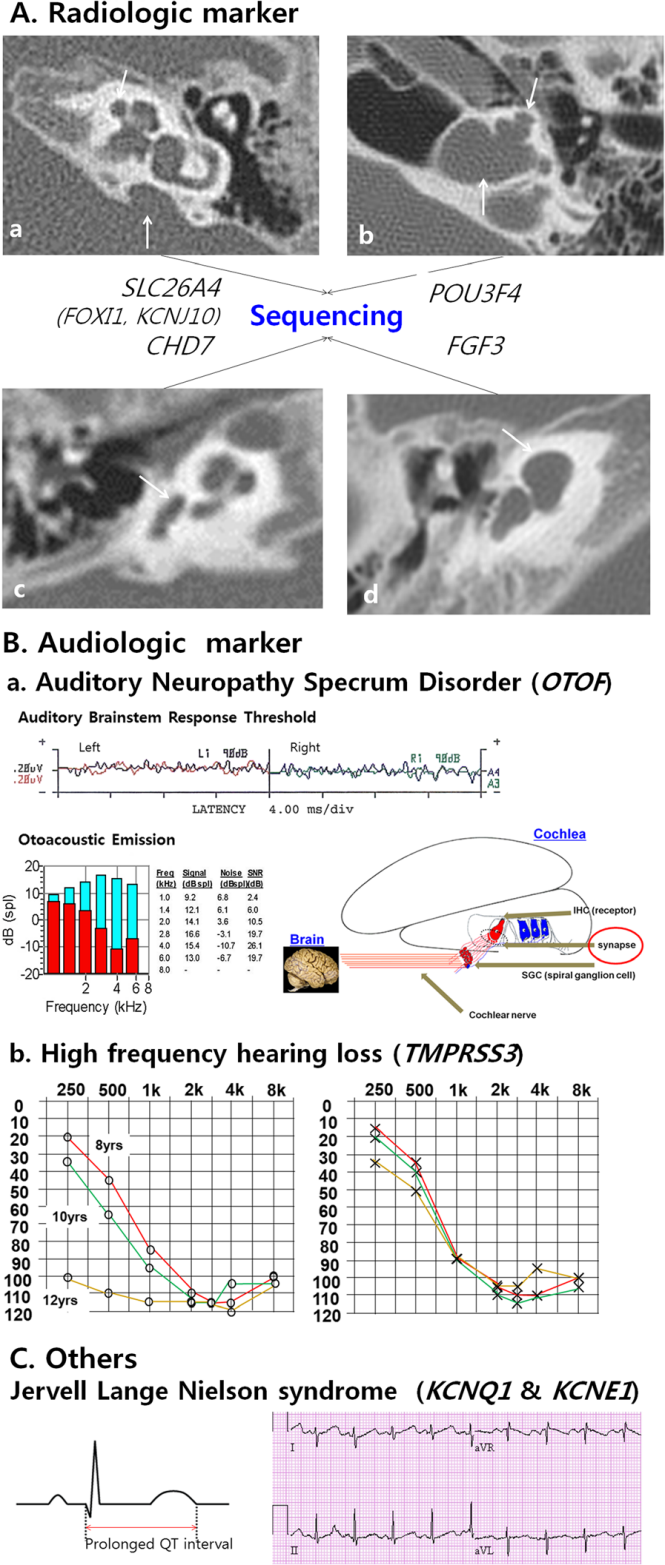
**Characteristic radiologic and audiologic markers. A**.(a) In cases with enlarged vestibular aqueducts (arrow head) with or without an incomplete partition defect (arrow) *SLC26A4* (with or without *FOXI1* and *KCNJ10*) was tested. (b) In cases with bulbous dilatation at the distal end of the IAC and basal turn of the cochlea incompletely separated from the IAC (arrow) were tested for *POU3F4*. (c) Patients suspected of CHARGE syndrome were tested for *CHD7*. The most common associated inner ear anomaly is semicircular canal dysplasia (arrow). (d) Sequencing of the *FGF3* gene was performed in patients with complete labyrinthine aplasia or cochlear hypoplasia (arrow). **B**.(a) Patients suspected of auditory neuropathy spectrum disorder were tested for the *OTOF* gene. The ‘otoacoustic emissions’ test is typically normal, whereas ‘auditory brainstem response’ is typically abnormal. No response was detected to 90 dB of click stimulus in the auditory brainstem response (upper panel). Signal-to-noise ratios of greater than 6 dB was shown in a specific frequency band in distortion product otoacoustic emissions (lower panel). (b) Audiograms showing ski slope type high frequency hearing loss with low frequency residual hearing initially and gradual deterioration with age. **C**. In cases of bilateral sensorineural hearing loss and a long EKG QT interval, *KCNQ1*/*KCNE1* was sequenced.

Genomic DNA was extracted from peripheral blood as previously described [21]. We previously established a new diagnostic pipeline combining PCR-based Sanger sequencing of phenotype driven candidate genes and targeted resequencing for testing familial hearing loss cases [20]. The approach was again implemented to make a molecular genetic diagnosis on cochlear implantees in this study (Figure 1). Subjects with either a characteristic radiologic marker or with a characteristic audiologic marker such as auditory neuropathy spectrum disorder or ski slope type high frequency hearing loss were directly subject to further Sanger sequencing of corresponding candidate genes (Figure 2) [22–31]. In cases with a long electrocardiographic QT interval (EKG), *KCNQ1*/*KCNE1* sequencing was performed. When there was no noticeable phenotypic marker, the *GJB2* gene was sequenced, as previously described [21]. In case of only one detectable mutant allele of *GJB2*, we performed breakpoint PCR to detect the reported large genomic deletions in the DFNB1 locus [32,33].

### Targeted resequencing of 204 deafness genes

Next, we applied targeted resequencing of known 204 deafness-related genes (TRS-204) for those without a potential pathogenic variant in candidate genes or the *GJB2* mutation. For targeted deep sequencing, customized baits were designed to capture all exons of 204 genes known to be associated with hearing loss or to be expressed in the inner ear in humans and/or mice (Additional file 1: Table S1). Genomic DNA was sequenced using Genome Analyzer II. Reads were aligned to the human genome reference sequence (hg19) using BWA-v0.7.5 with the ‘MEM’ algorithm. SAMTOOLS v0.1.18, GATK v2.4-7 and Picard v1.93 were used for processing SAM/BAM files, local realignment, and duplicate marking. Base recalibration was performed using GATK (known SNPs and indels from dbSNP137, Mills and 1000G gold standard indels b37 sites, and 1000G phase1 indels b37 sites). To identify mutations from the targeted genes, variants were called by Unified Genotyper in GATK and were also recalibrated by GATK based on dbSNP137, Mills indels, HapMap and Omni. The Perl script offered by ANNOVAR was used to annotate the variants. We firstly selected exonic and splicing variants including non-synonymous variants and small indels. Variants with allele frequency over 1% were discarded based on NHLBI-ESP 6500, 1000 Genome Project, and our in-house database consisting of exomes of 81 Korean individuals. We then included the variants that are not reported in dbSNP137. Low quality of reads (<20) and genotyping (<30) were then ruled out. We have finally prioritized the variants based on the inheritance pattern of deafness. Finally, we excluded the variants that we can detect from our 276 Korean normal hearing control chromosomes. A *trans* configuration of two detected variants was confirmed by checking parental samples in sporadic or autosomal recessive cases. Novel splice-site variants were considered pathogenic when they were not detected in the normal hearing control chromosomes and at the same time when they were predicted to cause significant changes that create or eliminate a splice-donor or splice acceptor site by ESEfinder (http://rulai.cshl.edu/cgi-bin/tools/ESE3/esefinder.cgi?process=home) and BDGP (http://www.fruitfly.org/seq_tools/splice.html).

We defined ‘highly probable cases’ as the ones with bi-allelic probable or possible pathogenic mutations in autosomal recessive or sporadic cases, or as cases with one probable or possible pathogenic mutation in autosomal dominant cases. We referred the autosomal recessive or sporadic cases with two mutations with one probably or possibly pathogenic mutation and an unknown variant in *trans* as ‘possibly explained cases’. Our detection rate was calculated to include both completely explained and possibly explained cases. Cases with only one definitely pathogenic mutation after TRS-204 was not considered as explained.

We have also performed CONTRA (COpy Number Targeted Resequencing Analysis) v2.0.4 and cn.MOPS (Copy Number estimation by a Mixture Of PoissonS) v1.8.0 using their default options, in order to detect possible copy number variation, such as, large deletions or duplications in targeted genes.

### Calculation of the sibling recurrence-risk for hearing loss

To gain insight of etiology and to estimate the risk of hearing loss recurrence, especially for families with an unknown etiology after TRS-204 (undiagnosed group), we calculated the sibling recurrence-risk for hearing loss. We used probands with a definitive autosomal recessive genotype as a control group (the control group). For this calculation, we additionally included one family (SNUH23), in which molecular genetic diagnosis was made during this study by only whole exome sequencing (not by TRS-204) due to family request. The sibling recurrence-risk was calculated using the segregation ratio of hearing loss among siblings of probands. We used Weinberg’s proband method to correct for possible ascertainment bias by excluding probands from the calculation as previously described [23,34]. In detail, an unbiased estimate of the segregation ratio (*P*) is calculated from the remaining members of the sibship:

$$ P=\frac{{\displaystyle \sum \left(r-1\right)}}{{\displaystyle \sum \left(s-1\right)}} $$

where *r* and *s* indicate the number of affected offspring and the total number of offspring in each family, respectively. Monozygotic twin pairs were treated as a single observation in the analysis as described above.

## Results

### Detection rate of phenotype driven candidate gene approach

At least one molecular genetic test was performed on each of the 93 probands who consented to genetic testing. Fifty four of the probands were male (58.0%) and 39 (41.9%) were female and overall mean age for CI was 8.9 years (10 months −72 years). Of the 93 probands, 42 who manifested either a characteristic inner ear abnormality or any remarkable auditory phenotype underwent direct Sanger sequencing of corresponding candidate genes. Molecular genetic diagnosis was successfully made in 30 (71.4%) of 42 subjects using this approach (Table 1 and Additional file 1: Table S2). All probands who underwent direct sequencing of *SLC26A4* (n = 13) and *POU3F4* (n = 5) for their enlarged vestibular aqueduct and incomplete partition type III, respectively turned out to carry pathogenic variants in the corresponding genes. Those who went through direct sequencing of *CHD7*, *OTOF*, and *KCNQ1* showed a detection rate of 77.7%, 20% and 100%, respectively. Therefore, the most rewarding phenotypic markers leading to molecular diagnosis were enlarged vestibular aqueduct (with/without incomplete partition type II) and incomplete partition type III, and long Q-T syndrome followed by lateral semicircular canal dysplasia as a part of the constellation of anomalies suggesting a CHARGE syndrome, and ski slope type high frequency hearing loss (Table 1). When we include only these five highly penetrant phenotypic markers for this approach, the detection rate of the causative mutation would rise up to 83.3% (30/36).

**Table 1:** **Phenotype-driven candidate gene approach and its detection rate of a causative mutation in cochlear implantees**

**Gene**	**Phenotype**	**Detection rate (n/N (%))**
***Radiologic marker***		
*SLC26A4*	AR, Enlarged vestibular aqueducts with or without Mondini deformity	13/13 (100%)
*CHD7*	AD, Charge syndrome (Mostly *de novo*)	7/9 (77.7%)
*POU3F4*	XR, Incomplete partition type III	5/5 (100%)
*FGF3*	AR, Complete labyrinthine aplasia or cochlear hypoplasia	0/2 (0%)
***Audiologic marker***		
*OTOF, Pejvakin*	AR, Auditory neuropathy spectrum disorder	1/5 (20%)
*TMPRSS3*	AR, Ski slope type high frequency hearing loss	3/7 (42.8%)
***Other characteristic marker***		
*KCNQ1 & KCNE1*	AR, Long QT syndrome	1/1(100%)
***Total***		**30/42 (71.4%)**

AD, autosomal dominant; AR, autosomal recessive; XR, X-linked recessive.

*GJB2* sequencing was performed as a screening test for 51 probands without any noticeable phenotypic marker. Ten subjects turned out to carry at least one mutant allele of *GJB2*, the prevalence of DFNB1 being 10.7% among the 93 cochlear implantees. Among these ten subjects, nine subjects carried two mutant alleles and one subject carried one mutant allele of p.R143W (Table 2).

**Table 2:** **Details of DFNB1 patients in our cohort with cochlear implantation**

**Patient**	**Gene**	**Inheritance**	**Mutation type**	**Gene bank No.**	**Chr**	**Exon**	**Nucleotide**	**Protein**	**dbSNP137/reference**
SHJ2	GJB2	Compound hetero	frameshift deletion frameshift deletion	NM_004004 NM_004004	13 13	2 2	c.176_191del c.235delC	p.Gly59Alafs×18 p.Leu79Cysfs×3	[35] rs80338943
SHJ12	GJB2	Compound hetero	nonsynonymous SNV nonsynonymous SNV	NM_004004 NM_004004	13 13	2 2	c.109G > A c.C427T	p.V37I p.R143W	rs72474224 rs80338948
SHJ18	GJB2	Homozygote	nonsynonymous SNV	NM_004004	13	2	c.C427T	p.R143W	rs80338948
SHJ31	GJB2	Compound hetero	frameshift deletion	NM_004004	13	2	c.176_191del	p.Gly59Alafs×18	[35]
			frameshift deletion	NM_004004	13	2	c.235delC	p.Leu79Cysfs×3	rs80338943
SHJ36	GJB2	Homozygote	frameshift deletion	NM_004004	13	2	c.35delG	p.Gly12Valfs×2	rs80338939
SHJ68	GJB2	Homozygote	frameshift deletion	NM_004004	13	2	c.235delC	p.Leu79Cysfs×3	rs80338943
SNUH60-136	GJB2	Single heterozygote	nonsynonymous SNV	NM_004004	13	2	c.C427T	p.R143W	rs80338948
SNUH70-160	GJB2	Compound hetero	frameshift deletion nonsynonymous SNV	NM_004004 NM_004004	13 13	2 2	c.235delC c.C427T	p.Leu79Cysfs×3 p.R143W	rs80338943 rs80338948
SNUH79-180	GJB2	Homozygote	frameshift deletion	NM_004004	13	2	c.235delC	p.Leu79Cysfs×3	rs80338943
SNUBH91-166	GJB2	Compound hetero	frameshift deletion nonsynonymous SNV	NM_004004 NM_004004	13 13	2 2	c.235delC c.C427T	p.Leu79Cysfs×3 p.R143W	rs80338943 rs80338948

Therefore, *GJB2* screening and the candidate gene approach based upon phenotype solved 43.0% (40/93) of the cases in terms of genetic etiology, leaving 53 probands (56.3%) unanswered in this population. These 53 probands included 12 in whom the candidate gene approach failed and 41 *GJB2* (−) probands without any phenotypic marker (Figure 1). These unsolved probands were subjected to the next step of our protocol, that is, targeted exome sequencing of known deafness related genes.

### Detection rate of TRS-204

**Figure 3:**
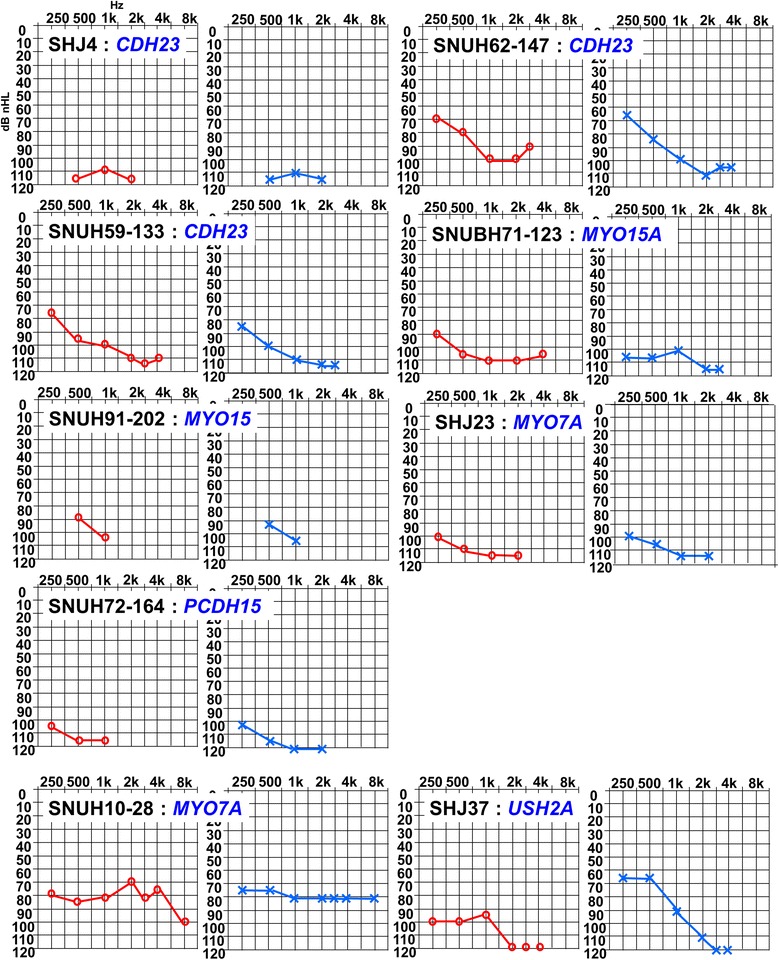
**Audiogram of probands who were genetically diagnosed by TRS-204.**

Due to their reluctance to TRS-204, eight of 53 probands dropped out after their initial screening where they did not get any confirmatory answer. Thus, 45 probands were available for the final analysis of targeted resequencing results. The detection rate of a causative mutation by TRS-204 for *GJB2*- negative cases without any characteristic phenotypic marker was 24.4% (11/ 45 subjects). Of these 11 with a positive genetic diagnosis, SB47-91 and SH3-7 were also subjected to TRS-80 and whole exome sequencing (WES) in addition to TRS-204, respectively. The TRS-80 and WES results of two (SB47-91 and SH3-7) were recently published separately [20,35,36], and the TRS-204 results of the eleven are presented (Table 3 and Figure 3). The most frequent causative gene revealed by this technology was *CDH23* (n = 3), followed by *MYO15A* (n = 2), *MYO7A* (n = 2) and other four deafness genes (*PCDH15* (n = 1), *USH2A* (n = 1), *MYO3A* (n = 1) and *ACTG1* (n = 1)) (Table 3). Of these the 11 cases, 9 were ‘highly probable cases’ carrying mutations in one of the 204 deafness genes and two cases were classified as possibly explained (Table 3). A *trans* configuration of the variants in these two possibly explained cases were confirmed by checking parental samples, and the four missense variants in these two cases were not detected in 276 normal hearing control chromosomes. Rigorous ophthalmological examinations have not revealed any abnormality from SHJ4, SHJ23, SNUH59-133, SH62-147, SNUH72-164, and SNUH10-28, making these cases carrying mutations of USH1genes to be non-syndromic. Lack of ophthalmologic abnormal finding from SHJ37 carrying *USH2A* variants does not rule out a possibility of USH2 since retinitis pigmentosa can develop later. There were also three probands (SHJ3, SHJ16, and SNUH53-118) with only one definitely pathogenic mutant allele in one of three recessive deafness genes, *OTOA*, *MYO15A*, and *MYO7A,* respectively (Table 3). However, we were not able to detect any potentially pathogenic allele in the coding region in a *trans* configuration with the mutation in these three probands, making these cases unanswered in terms of molecular genetic etiology.

**Table 3:** **Details of final candidates from eleven deaf subjects molecular genetically diagnosed and three subjects with only one mutant allele of recessive genes after targeted exome sequencing of 204 deafness genes**

**A) Highly probable cases (n=9)**														
**Patient**	**Gene**	**Inheritance**	**DP**	**GQ**	**Type**	**Chr**	**Genbank No.**	**Exon**	**Nucleotide**	**Protein**	**GERP++**	**PolyPhen2**	**Reference**	**Control**
SHJ4	CDH23	Homo (AR)	218	99	nonsynonymous SNV	Chr10	NM_022124	Exon8	c.C719T^*^	p.P240L^*^	5.19	Probably damaging	rs1219083 (flagged)*	
SHJ23	MYO7A	Compound hetero (AR)	82	99	Stopgain SNV	Chr11	NM_000260	Exon 3	c.C52T	p.Q18X	3.91	NA	This study	
			178	99	Stopgain SNV	Chr11	NM_000260	Exon 18	c.C2115A	p.C705X	3.03	NA	This study	
SNUH59-133 (SHJ33)	CDH23	Compound hetero (AR)	238	99	nonsynonymous SNV	Chr10	NM_022124	Exon 8	c.C719T*	p.P240L*	5.19	Probably damaging	rs1219083 (flagged)^§^	
			29	99	nonsynonymous SNV	Chr10	NM_022124	Exon 37	c.C4853A	p.T1618K	5.9	Probably damaging	This study	0/276
SH62-147 (SHJ41)	CDH23	Compound hetero (AR)	178	99	nonsynonymous SNV	Chr10	NM_022124	exon42	c.G5747A	p.R1916H	4.28	Probably damaging	This study	0/276
			237	99	nonsynonymous SNV	Chr10	NM_022124	exon46	c.G6604A^§^	p.D2202N^§^	5.06	Probably damaging	rs121908349 (flagged)	
SNUH72-164 (SHJ52)	PCDH15	Compound hetero (AR)	238	99	nonsynonymous SNV	Chr10	NM_001142769	exon36	c.5035G > C	p.V1679L	3.48	Damaging^***^	This study	0/276
			250	99	Frameshift deletion	Chr10	NM_001142763	exon23	c.2927delA	p.Gln976Argfs*18	N/A	NA	This study	
SNUH91-202 (SHJ70)	MYO15A	Compound hetero (AR)	76	99	Stopgain SNV	Chr17	NM_016239	exon2	c.G535T	p.E179X	5.48	NA	This study	
			147	99	nonsynonymous SNV	Chr17	NM_016239	exon10	c.G4252A	p.G1418R	5.31	Probably damaging	This study	0/276
SNUBH71-123	MYO15A	Compound hetero (AR)	101	99	Splice donor variant	Chr17	NM_016239	exon10	c.4320 + 1G > A		5.84	NA	This study	
			70	99	nonsynonymous SNV	Chr17	NM_016239	exon46	c.T8396A	p.L2799H	5.58	Probably damaging	This study	0/276
SNUBH47-91**	MYO3A	Compound hetero (AR)	100	99	nonsynonymous	Chr10	NM_017433	exon7	c.C580A	p.P194T	4.30	Probably damaging	[20]	
			77	99	Frameshift insetion	Chr10	NM_017433	exon16	c.1582_1583 insT	p.Y530Lfs*9	N/A	N/A	[20]	
SNUH3-7**	ACTG1	Singe hetero(AD)	30	99	nonsynonymous	Chr17	NM_001199954	exon5	c.T914C	p.M305T	4.05	Probably damaging	[36]	
**B) Possibly explained cases (n=2)**														
SNUH10-28	MYO7A	Compound hetero (AR)	7	95	nonsynonymous SNV	Chr11	NM_000260	exon23	c.C2724G	p.D908E	-8.53	Benign	This study	0/276
			10	85	nonsynonymous SNV	Chr11	NM_000260	exon29	c.C3701G	p.T1234S	5.38	Possibly damaging	This study	0/276
SHJ37	USH2A	Compound hetero (AR)	238	99	nonsynonymous SNV	Chr1	NM_206933	exon64	c.T14017C	p.Y4673H	5.09	Probably damaging	This study	0/276
			237	99	nonsynonymous SNV	Chr1	NM_206933	exon2	c.C419A	p.P140H	2.26	Benign	This study	0/276
**C) Cases with only one probably pathogenic recessive allele (n=3)**														
SHJ3	OTOA	AR	250	99	Frameshift	Chr16	NM_170664, NM_001161683, NM_144672	exon7, exon12, exon16	c.792delC, c.1527delC, c.1764delC	p.Gln589Argfs*55	NA	NA	This study	
SHJ16	MYO15A	AR	72	99	Splice site	Chr17	NM_016239	exon4	c.3756+1G>A	NA	4.19	NA	[37]	
SNUH53-118	MYO7A	AR	185	99	Stopgain	Chr11	NM_000260	exon19	c.C2254T	p.Q752X	5.03	NA	This study	

*rs1219083(flagged);^§^rs121908349(flagged).

**SNUBH47-91 and SNUH3-7 was also subjected to TRS-80 and WES in addition to TRS-204, respectively, and the result of TRS-80 and WES was recently published separately [20,36].

***DAMAGING by SIFT, PolyPhen2 result: Unknown WARNING: BLAST search results for PCDH15 truncated due to total number of HSPs >2000.

**Figure 4:**
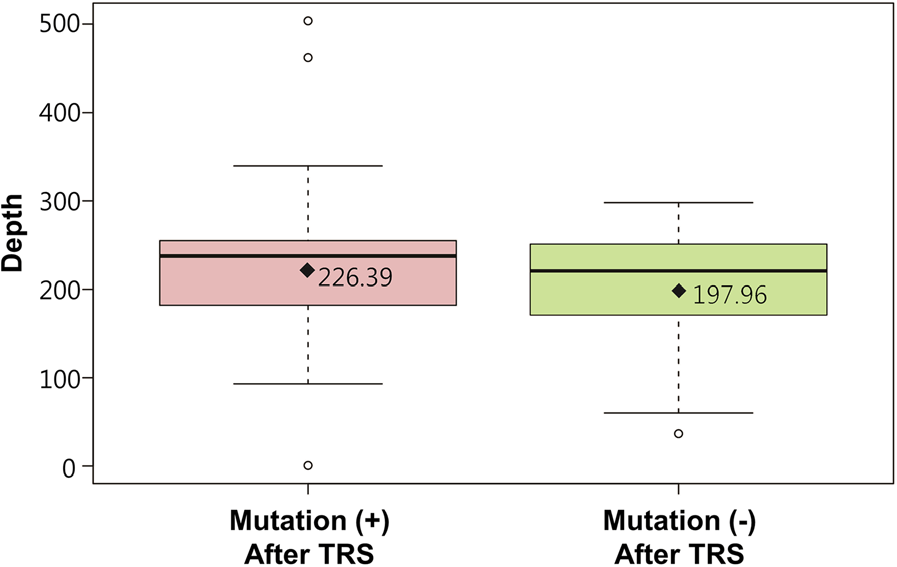
**Boxplot for average mean depth.** Black dot represents average mean depth for solved and unsolved cases.

To exclude a possibility of low coverage of unsolved cases by TRS-204, we compared the coverage of solved cases after TRS-204 with that of unsolved cases. Average mean depths were 226.39 and 197.96 for solved and unsolved cases, respectively (Additional file 1: Table S3 and Figure 4). A two tailed Student’s t-test assuming unequal variances was used to determine whether there existed a significant difference. The critical value of 2.09 was greater than the absolute value of the test statistic (0.94), which means there was no statistically significant difference in the coverage between the solved and unsolved groups by TRS-204.

Regarding copy number variation (CNV), we have not detected any deletion or duplication on the TRS-204 genes targeted, based on the results from CONTRA and cn.MOPS. We, in particular, focused upon the regions covering *OTOA*, *MYO15A*, and *MYO7A* from three probands with only one detectable mutant allele in one of these recessive genes to exclude any CNV possibly in a *trans* configuration with the mutant alleles. However, we have confirmed that there was no detectable CNV.

### Overall Efficacy of our hierarchical molecular genetic test protocol for cochlear implantees

Overall, monogenic Mendelian etiology was documented in 51 (54.8%) of the 93 Korean cochlear implantees who decided to participate in this study (Figure 1). Mutations in *SLC26A4* and *GJB2* accounted for 24.7% (23/93) of the 93 cochlear implantees, and the incorporation of TRS-204 into our protocol increased the detection rate by 11.8% (11/93) in this population.

### The sibling recurrence-risk of hearing loss among the siblings of probands

**Figure 5:**
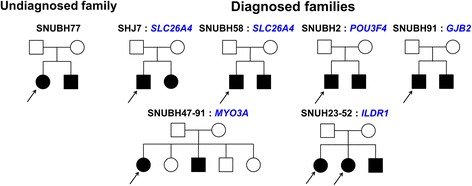
**Pedigrees of multiplex families that were employed for calculation of the sibling recurrence risk in this study.** Simplex families were not described here. A black arrow indicates the affected sibling that contributes to the recurrence risk.

Total 42 families including 34 families with an unknown etiology after TRS-204 were designated the undiagnosed group. Of the 52 families (including one family (SNUH18) with a genetic diagnosis obtained by direct application of whole exome sequencing during this study period) with a definitive genetic diagnosis, 8 families that carried an autosomal dominant mutation from *CHD7* or *ACTG1* were excluded, leaving a total number of 44 control families only with recessive inheritance. Therefore, a total of 38 siblings from 42 undiagnosed families and 37 siblings from the 44 control families were reevaluated. Pedigrees of multiplex families were provided (Figure 5).

The sibling recurrent-risk of deafness among siblings of the undiagnosed group (0.03 (1/38)) was significantly lower than that (0.19 (7/37)) of the control group, which showed an autosomal recessive inheritance pattern (p = 0.028, Fisher’s exact test (two tailed)) (Table 4). The risk of hearing loss recurrence in the control group with a definitive genetic diagnosis ranged from 0.09 to 0.34 for a 95% confidence interval. This range was compatible with autosomal recessive inheritance because 0.25 fell within the mid range of this interval. In contrast, the 95% confidence interval of the risk of hearing loss recurrence among siblings of the undiagnosed group ranged from 0 to 0.13. This excluded 0.25, the ratio indicative of monogenic autosomal recessive inheritance or digenic inheritance, thereby making it unlikely that hearing loss in the undiagnosed group exhibited such inheritance patterns (Table 4).

**Table 4:** **Recurrence risk (segregation ratio) of hearing loss among siblings**

**Molecular genetic status**	**Segregation ratio**	**95% CI** ^*^
**Undiagnosed** (N = 42)	1/38 (0.03)^**^	0-0.12
**Control** (N =44)	7/37 (0.19)^**^	0.09-0.34

CI, confidence interval, ^**^p = 0.028 by Fisher’s exact test (two tailed).

## Discussion

More than 400 genetic syndromes are associated with hearing loss. More than 140 genetic loci associated with nonsyndromic hearing loss have been mapped and more than 60 genes identified (http://hereditaryhearingloss.org/). An etiologic diagnosis of deafness can be useful for determining prognosis (i.e., whether the loss will worsen), optimal intervention (e.g., hearing aids, CI, sign language), and recurrence risks of hearing loss for future children and other family members [9]. In particular, from the prognostic perspective, we can expect successful results of CI in hereditary deafness with mutation of specific genes, such as, *GJB2*, *SLC26A4*, mitochondrial mutations, *OTOF*, Usher syndrome type I, *DFNA9* (*COCH*), and *DFNA17* (*MYH9*) [6]. Therefore, genetic testing has become a crucial component of the diagnostic work-up for cochlear implant candidates from etiologic and prognostic perspectives. The main deafness genes and their mutation spectra depend upon ethnicity [38]. However, few comprehensive studies have been conducted using molecular genetic testing and epidemiology for a larger cohort of cochlear implantees who mainly but comprised sporadic deafness cases, not consanguineous multiplex cases. In this regard, this study merits special attention. By incorporating the largest deafness panel in the literature for sporadic cochlear implantees to our protocol, we clearly documented the molecular etiology and the genotypes of about half of such cases, beyond just estimating the proportion of genetic hearing loss. Brownstein et al. (2011) used a panel targeting 246 deafness genes, however, their cohort mainly comprised multiplex families [19].

In the NGS era, the candidate gene approach retains its value as a first line test for the molecular genetic diagnosis of deafness in Korean population [20]. This approach is easily applied and cost effective when there is sufficient knowledge about the expressed phenotype and biological functional impact of the genetic alteration present. In this study, we expanded the use of this approach to include seven phenotypic markers. Based upon the result obtained, we refined the candidate gene approach to focus more upon phenotypes because they provide better clues regarding effective molecular genetic diagnosis. In our Korean population, five phenotypic markers were especially rewarding, and the detection rate of this approach driven by these five phenotypic markers reached 83.3%. Furthermore, enlarged vestibular aqueduct, incomplete partition type III, lateral canal dysplasia as a manifestation of CHARGE syndrome was a very highly penetrant feature of mutations in *SLC26A4*, *POU3F4*, and *CHD7*, respectively in this population. *TMPRSS3* also merits attention due to its relatively high prevalence (3 cases) when focusing upon the CI implantees initially manifesting post lingual ski slope type hearing loss. Note that mutations of this gene were rarely detected in congenital profound deafness in this population [18,39]. Finally, sequencing of *OTOF* in the auditory neuropathy spectrum disorder (ANSD) led to molecular genetic diagnosis only in one of five CI cases with ANSD, calling the usefulness of this approach for ANSD into question at least for CI candidates/implantees in this population.

The prevalences of the mutation in implanted subjects seem to be dependent on ethnicities. For DFNB1, which has been considered the most important known cause of non-syndromic autosomal recessive deafness in Caucasians, a high occurrence of *GJB2* mutations among cochlear implantees was reported in Romania, Portugal, and Slovakia [1,40,41], in which it reach over one-third of all investigated probands. On the other hand, much lower (<15 %) contributions of DFNB1 mutations have been reported in Turkey [42], USA (Baltimore) [43] and Korea [44]. The prevalence of DFNB1, 10.7%, among total 93 cochlear implantees in the present study concurs with that found in a previous Korean study [44]. In our current study, the detection rate of *SLC26A4* mutations was noted to be 14% which is higher than that of *GJB2*. This figure is very similar with those reported in previous Korean studies, again supporting a significant contribution of alteration of *SLC26A4* to hearing loss in this population [44,45].

Regarding the molecular genetic diagnosis of *GJB2* negative cases without any characteristic phenotypic marker, TRS-204 can provide a convincing answer in one fourth of such cases. Our TRS-204 data revealed that *CDH23*, *MYO15A,* and *MYO7A* constituted the majority of causative genes, whereas other genes, that is, *PCDH15*, *USH2A*, *MYO3A*, and *ACTG1* were each detected in only one patient. Furthermore, this pattern is similar to that reported in Japanese populations [46]. Checking the audiograms of the probands who were genetically diagnosed by TRS-204, there was no remarkable finding in terms of the degree and configuration of residual hearing that we can possibly relate to the mutation of a certain gene (Figure 3). The usefulness of TRS-204 can be further emphasized in this regard in Koreans. Correspondingly, about 54.8% of Korean cochlear implantees who willingly participated in the molecular genetic test were clearly explained by the monogenic Mendelian disorder definitely involving one of two hundred deafness-related genes, when our hierarchical approach combining phenotype-driven candidate gene sequencing and TRS-204 was applied. This proportion is just slightly higher than a previous estimate (51%) for a single gene disorder in profound childhood deafness [47]. This similarity is remarkable considering the variations in marriage rates and patterns of deafness and sibship sizes between populations. In previous studies, families with consanguineous marriages and large sibships were preferentially recruited [47]. Although the majority of our deaf probands were singleton (sporadic) cases, monogenic Mendelian disorder was documented in more than half of our cases. Therefore, when singleton cases of profound hearing loss are encountered in the Korean population, it can be presumed that deafness is due to a monogenic Mendelian genetic disorder at least in half of cases.

Discovery of a specific genetic etiology in a significant portion of presumed autosomal recessive or even sporadic hearing loss cases by targeted sequencing was recently reported by others [3,48]. Shearer et al. (2013) identified specific genetic etiologies in 11 of 32 sporadic hearing loss cases (34%) and 22 of 49 presumed recessive hearing loss cases (56%) by deep sequencing of 60 deafness genes, but this cohort included variable degrees of hearing loss. Additionally, in a Japanese study, 37.5% (81/216) of deafness cases were molecular genetically diagnosed by the targeted exon resequencing of 112 deafness genes [3], but the cohort also included variable degrees of hearing loss, which could account for differences between their detection rate and that observed in the present study. It is also possible that there was greater recruitment bias toward a higher prevalence of CHARGE syndrome (n = 7) and incomplete partition type III (n = 5) with highly penetrant marker of *CHD7* and *POU3F4* mutations, in our CI cohort than in the Japanese cohort, as many deaf subjects with such severe inner anomaly in Korea tend to visit our tertiary referral center.

In our study, only 93 subjects participated in the molecular genetic test among initially eligible 236 patients. The low participation rate can be attributed to the policy of reimbursement by some private insurance companies against genetically documented cases in Korea and also to the Korean culture where there is reluctance of parents to disclose that hearing loss of their children is genetic. Additionally, those who live in a far located province were also reluctant to make an extra visit to participate in the test. As for the large drop out, we do not think that there was a significant recruitment bias either toward or against genetic cases for the following reasons. Firstly, there was no difference in the proportion of multiplex hearing loss families between the participants and non-participants. The majority of families were singleton cases for both groups. Secondly, the frequency of mutations in *GJB2* and *SLC26A4* in this study did not differ from what was previously reported in the Korean population, suggesting the drop out in this study was not significantly biased.

We also sought to determine the etiologies of subjects without a candidate causative variant detected by TRS-204, and to determine whether these cases were non-genetic or non-Mendelian. It is also possible causative genes resided beyond the 204 target deafness genes or that true causative genes were not sufficiently covered by TRS-204. In addition, CNVs might play a role in these cases. Initially, we compared the depth of coverage of target genes between cases with and without candidate variants, but found similar target gene coverages in these two groups (Figure 4). Therefore, we were able to minimize the possibility that poor coverage of target genes was responsible for a failure to detect candidate variants. In addition, we checked our TRS-204 data to see if there was any deletion or duplication of targeted genes. However, we did not detect any convincing CNV that could account for deafness in our cohort. Finally, we calculated the recurrent risk of deafness among siblings from families without any candidate variant after TRS-204. The most striking observation in this study was a significantly lower risk of hearing loss recurrence (0.03 (1/38)) in the undiagnosed group than in the control group segregating autosomal recessive deafness. The 95% confidence interval of the recurrence rate of hearing loss from our undiagnosed group did not include 0.25, the ratio indicative of autosomal recessive inheritance. Our findings indicate monogenic Mendelian disorder caused by mutations in genes beyond the 204 target genes did not account for the etiology of a substantial portion of the undiagnosed group after TRS-204 in the Korean population.

In this regard, our findings also call the usefulness of whole exome sequencing into question in this undiagnosed group after TRS-204 testing in Koreans. Recently, whole exome sequencing has substantially aided the detection of a series of new causative deafness genes [49–55]. However, it was more successful in consanguineous families with multiple affected members. Moreover, most of the families included had a significant linkage interval, which indicated a Mendelian monogenic disorder and thereby enabled whole exome sequencing of genes only within limited intervals. In contrast, our cochlear implantees not diagnosed by TRS-204 in the Korean population were mostly singleton cases without any linkage intervals. Undiagnosed oligogenic/multigenic disorder, complex trait, or a non-genetic etiology, such as, non-symptomatic CMV infection [56] might have contributed to the pathogeneses of our undiagnosed cases.

## Conclusion

When our hierarchical genetic test protocol was applied, Mendelian monogenic disorder can explain about half of CI implantees who willingly participated in genetic tests in the Korean population where the majority of cases were sporadic. In addition, a substantial proportion of undiagnosed sporadic cases can be viewed to have other than a monogenic autosomal recessive/digenic etiology. This finding should be considered during further genetic test protocol planning for the undiagnosed cases and when counseling these families.

## Additional file

Additional file 1: Table S1. 204 deafness genes. **Table S2.** Details of variants in *SLC26A4*, *POU3F4*, *OTOF* and *KCNQ1* detected from the phenotype driven candidate gene approach. **Table S3.** Summary statistics of targeted exome sequencing for 45 samples [57–60].

## Abbreviations

CI: Cochlear implantation
CONTRA: Copy number targeted resequencing analysis
cn.MOPS: Copy number estimation by a mixture of poissons
CNV: Copy number variation
IRB: Institutional review boards
NGS: Next generation sequencing
SNHL: Sensorineural hearing loss
TRS: Targeted resequencing
TRS-204: Targeted resequencing method of 204 candidate deafness genes

## References

[1] Varga L, Masindova I, Huckova M, Kabatova Z, Gasperikova D, Klimes I, Profant M (2014). Prevalence of DFNB1 mutations among cochlear implant users in Slovakia and its clinical implications. Eur Archives Otorhinolaryngol.

[2] Wu CC, Liu TC, Wang SH, Hsu CJ, Wu CM (2011). Genetic characteristics in children with cochlear implants and the corresponding auditory performance. Laryngoscope.

[3] Miyagawa M, Naito T, Nishio SY, Kamatani N, Usami S (2013). Targeted exon sequencing successfully discovers rare causative genes and clarifies the molecular epidemiology of Japanese deafness patients. PLoS One.

[4] Joshi VM, Navlekar SK, Kishore GR, Reddy KJ, Kumar EC (2012). CT and MR imaging of the inner ear and brain in children with congenital sensorineural hearing loss. Radiographics.

[5] Elziere M, Roman S, Nicollas R, Triglia JM (2012). Value of systematic aetiological investigation in children with sensorineural hearing loss. Eur Ann Otorhinolaryngol Head Neck Dis.

[6] Wu CC, Lee YC, Chen PJ, Hsu CJ (2008). Predominance of genetic diagnosis and imaging results as predictors in determining the speech perception performance outcome after cochlear implantation in children. Arch Pediatr Adolesc Med.

[7] Kabatova Z, Profant M, Simkova L, Groma M, Nechojdomova D (2009). Cochlear implantation in malformed inner ear. Bratisl Lek Listy.

[8] Black J, Hickson L, Black B, Perry C (2011). Prognostic indicators in paediatric cochlear implant surgery: a systematic literature review. Cochlear Implants Int.

[9] Brown KK, Rehm HL (2012). Molecular diagnosis of hearing loss. Curr Protoc Hum Genet.

[10] Scott DA, Carmi R, Elbedour K, Yosefsberg S, Stone EM, Sheffield VC (1996). An autosomal recessive nonsyndromic-hearing-loss locus identified by DNA pooling using two inbred Bedouin kindreds. Am J Hum Genet.

[11] Yuan Y, You Y, Huang D, Cui J, Wang Y, Wang Q, Yu F, Kang D, Yuan H, Han D, Dai P (2009). Comprehensive molecular etiology analysis of nonsyndromic hearing impairment from typical areas in China. J Transl Med.

[12] Lin X, Tang W, Ahmad S, Lu J, Colby CC, Zhu J, Yu Q (2012). Applications of targeted gene capture and next-generation sequencing technologies in studies of human deafness and other genetic disabilities. Hear Res.

[13] Rehman AU, Morell RJ, Belyantseva IA, Khan SY, Boger ET, Shahzad M, Ahmed ZM, Riazuddin S, Khan SN, Riazuddin S, Friedman TB (2010). Targeted capture and next-generation sequencing identifies C9orf75, encoding taperin, as the mutated gene in nonsyndromic deafness DFNB79. Am J Hum Genet.

[14] Walsh T, Shahin H, Elkan-Miller T, Lee MK, Thornton AM, Roeb W, Abu Rayyan A, Loulus S, Avraham KB, King MC, Kanaan M (2010). Whole exome sequencing and homozygosity mapping identify mutation in the cell polarity protein GPSM2 as the cause of nonsyndromic hearing loss DFNB82. Am J Hum Genet.

[15] Shearer AE, DeLuca AP, Hildebrand MS, Taylor KR, Gurrola J, Scherer S, Scheetz TE, Smith RJ (2010). Comprehensive genetic testing for hereditary hearing loss using massively parallel sequencing. Proc Natl Acad Sci U S A.

[16] Pierce SB, Walsh T, Chisholm KM, Lee MK, Thornton AM, Fiumara A, Opitz JM, Levy-Lahad E, Klevit RE, King MC (2010). Mutations in the DBP-deficiency protein HSD17B4 cause ovarian dysgenesis, hearing loss, and ataxia of Perrault Syndrome. Am J Hum Genet.

[17] Zheng J, Miller KK, Yang T, Hildebrand MS, Shearer AE, DeLuca AP, Scheetz TE, Drummond J, Scherer SE, Legan PK, Goodyear RJ, Richardson GP, Cheatham MA, Smith RJ, Dallos P (2011). Carcinoembryonic antigen-related cell adhesion molecule 16 interacts with alpha-tectorin and is mutated in autosomal dominant hearing loss (DFNA4). Proc Natl Acad Sci U S A.

[18] Baek JI, Oh SK, Kim DB, Choi SY, Kim UK, Lee KY, Lee SH (2012). Targeted massive parallel sequencing: the effective detection of novel causative mutations associated with hearing loss in small families. Orphanet J Rare Dis.

[19] Brownstein Z, Friedman LM, Shahin H, Oron-Karni V, Kol N, Abu Rayyan A, Parzefall T, Lev D, Shalev S, Frydman M, Davidov B, Shohat M, Rahile M, Lieberman S, Levy-Lahad E, Lee MK, Shomron N, King MC, Walsh T, Kanaan M, Avraham KB (2011). Targeted genomic capture and massively parallel sequencing to identify genes for hereditary hearing loss in Middle Eastern families. Genome Biol.

[20] Choi BY, Park G, Gim J, Kim AR, Kim BJ, Kim HS, Park JH, Park T, Oh SH, Han KH, Park WY (2013). Diagnostic application of targeted resequencing for familial nonsyndromic hearing loss. PLoS One.

[21] Kim SY, Park G, Han KH, Kim A, Koo JW, Chang SO, Oh SH, Park WY, Choi BY (2013). Prevalence of p.V37I variant of GJB2 in mild or moderate hearing loss in a pediatric population and the interpretation of its pathogenicity. PLoS One.

[22] Pryor SP, Madeo AC, Reynolds JC, Sarlis NJ, Arnos KS, Nance WE, Yang Y, Zalewski CK, Brewer CC, Butman JA, Griffith AJ (2005). SLC26A4/PDS genotype-phenotype correlation in hearing loss with enlargement of the vestibular aqueduct (EVA): evidence that Pendred syndrome and non-syndromic EVA are distinct clinical and genetic entities. J Med Genet.

[23] Choi BY, Madeo AC, King KA, Zalewski CK, Pryor SP, Muskett JA, Nance WE, Butman JA, Brewer CC, Griffith AJ (2009). Segregation of enlarged vestibular aqueducts in families with non-diagnostic SLC26A4 genotypes. J Med Genet.

[24] Yang T, Gurrola JG, Wu H, Chiu SM, Wangemann P, Snyder PM, Smith RJ (2009). Mutations of KCNJ10 together with mutations of SLC26A4 cause digenic nonsyndromic hearing loss associated with enlarged vestibular aqueduct syndrome. Am J Hum Genet.

[25] Yang T, Vidarsson H, Rodrigo-Blomqvist S, Rosengren SS, Enerback S, Smith RJ (2007). Transcriptional control of SLC26A4 is involved in Pendred syndrome and nonsyndromic enlargement of vestibular aqueduct (DFNB4). Am J Hum Genet.

[26] Lee HK, Lee SH, Lee KY, Lim EJ, Choi SY, Park RK, Kim UK (2009). Novel POU3F4 mutations and clinical features of DFN3 patients with cochlear implants. Clin Genet.

[27] Song MH, Cho HJ, Lee HK, Kwon TJ, Lee WS, Oh S, Bok J, Choi JY, Kim UK (2011). CHD7 mutational analysis and clinical considerations for auditory rehabilitation in deaf patients with CHARGE syndrome. PLoS One.

[28] Tekin M, Hismi BO, Fitoz S, Ozdag H, Cengiz FB, Sirmaci A, Aslan I, Inceoglu B, Yuksel-Konuk EB, Yilmaz ST, Yasun O, Akar N (2007). Homozygous mutations in fibroblast growth factor 3 are associated with a new form of syndromic deafness characterized by inner ear agenesis, microtia, and microdontia. Am J Hum Genet.

[29] Tekin M, Ozturkmen Akay H, Fitoz S, Birnbaum S, Cengiz FB, Sennaroglu L, Incesulu A, Yuksel Konuk EB, Hasanefendioglu Bayrak A, Senturk S, Cebeci I, Utine GE, Tunçbilek E, Nance WE, Duman D (2008). Homozygous FGF3 mutations result in congenital deafness with inner ear agenesis, microtia, and microdontia. Clin Genet.

[30] Riazuddin S, Ahmed ZM, Hegde RS, Khan SN, Nasir I, Shaukat U, Riazuddin S, Butman JA, Griffith AJ, Friedman TB, Choi BY (2011). Variable expressivity of FGF3 mutations associated with deafness and LAMM syndrome. BMC Med Genet.

[31] Weegerink NJ, Schraders M, Oostrik J, Huygen PL, Strom TM, Granneman S, Pennings RJ, Venselaar H, Hoefsloot LH, Elting M, Cremers CW, Admiraal RJ, Kremer H, Kunst HP (2011). Genotype-phenotype correlation in DFNB8/10 families with TMPRSS3 mutations. J Assoc Res Otolaryngol.

[32] del Castillo FJ, Rodriguez-Ballesteros M, Alvarez A, Hutchin T, Leonardi E, de Oliveira CA, Azaiez H, Brownstein Z, Avenarius MR, Marlin S, Pandya A, Shahin H, Siemering KR, Weil D, Wuyts W, Aguirre LA, Martín Y, Moreno-Pelayo MA, Villamar M, Avraham KB, Dahl HH, Kanaan M, Nance WE, Petit C, Smith RJ, Van Camp G, Sartorato EL, Murgia A, Moreno F, del Castillo I (2005). A novel deletion involving the connexin-30 gene, del(GJB6-d13s1854), found in trans with mutations in the GJB2 gene (connexin-26) in subjects with DFNB1 nonsyndromic hearing impairment. J Med Genet.

[33] Lerer I, Sagi M, Ben-Neriah Z, Wang T, Levi H, Abeliovich D (2001). A deletion mutation in GJB6 cooperating with a GJB2 mutation in trans in non-syndromic deafness: a novel founder mutation in Ashkenazi Jews. Hum Mutat.

[34] Cho SW, Kang SI, Park SJ, Kim AR, Koo JW, Kim CS, Lee JH, Chang SO, Oh SH, Choi BY (2013). Clinical characteristics of patients with narrow bony cochlear nerve canal: is the bilateral case just a duplicate of the unilateral case?. Laryngoscope.

[35] Kudo T, Ikeda K, Kure S, Matsubara Y, Oshima T, Watanabe K, Kawase T, Narisawa K, Takasaka T (2000). Novel mutations in the connexin 26 gene (GJB2) responsible for childhood deafness in the Japanese population. Am J Med Genet.

[36] Park G, Gim J, Kim AR, Han KH, Kim HS, Oh SH, Park T, Park WY, Choi BY (2013). Multiphasic analysis of whole exome sequencing data identifies a novel mutation of ACTG1 in a nonsyndromic hearing loss family. BMC Genomics.

[37] Liburd N, Ghosh M, Riazuddin S, Naz S, Khan S, Ahmed Z, Riazuddin S, Liang Y, Menon PS, Smith T, Smith AC, Chen KS, Lupski JR, Wilcox ER, Potocki L, Friedman TB (2001). Novel mutations of MYO15A associated with profound deafness in consanguineous families and moderately severe hearing loss in a patient with Smith-Magenis syndrome. Hum Genet.

[38] Propst EJ, Stockley TL, Gordon KA, Harrison RV, Papsin BC (2006). Ethnicity and mutations in GJB2 (connexin 26) and GJB6 (connexin 30) in a multi-cultural Canadian paediatric Cochlear Implant Program. Int J Pediatr Otorhinolaryngol.

[39] Chung J, Park SM, Chang SO, Chung T, Lee KY, Kim AR, Park JH, Kim V, Park WY, Oh SH, Kim D, Park WJ, Choi BY: **A novel mutation of TMPRSS3 related to milder auditory phenotype in Korean postlingual deafness: a possible future implication for a personalized auditory rehabilitation.** *J Mol Med* 2014, 10.1007/s00109-014-1128-3 24526180

[40] Radulescu L, Martu C, Birkenhager R, Cozma S, Ungureanu L, Laszig R (2012). Prevalence of mutations located at the dfnb1 locus in a population of cochlear implanted children in eastern Romania. Int J Pediatr Otorhinolaryngol.

[41] Chora JR, Matos TD, Martins JH, Alves MC, Andrade SM, Silva LF, Ribeiro CA, Antunes MC, Fialho MG, Caria MH (2010). DFNB1-associated deafness in Portuguese cochlear implant users: prevalence and impact on oral outcome. Int J Pediatr Otorhinolaryngol.

[42] Tarkan O, Sari P, Demirhan O, Kiroglu M, Tuncer U, Surmelioglu O, Ozdemir S, Yilmaz MB, Kara K (2013). Connexin 26 and 30 mutations in paediatric patients with congenital, non-syndromic hearing loss treated with cochlear implantation in Mediterranean Turkey. J Laryngol Otol.

[43] Lustig LR, Lin D, Venick H, Larky J, Yeagle J, Chinnici J, Polite C, Mhatre AN, Niparko JK, Lalwani AK (2004). GJB2 gene mutations in cochlear implant recipients: prevalence and impact on outcome. Arch Otolaryngol Head Neck Surg.

[44] Shin JW, Lee SC, Lee HK, Park HJ (2012). Genetic screening of GJB2 and SLC26A4 in Korean cochlear implantees: experience of soree Ear clinic. Clin Exp Otorhinolaryngol.

[45] Lee KY, Choi SY, Bae JW, Kim S, Chung KW, Drayna D, Kim UK, Lee SH (2008). Molecular analysis of the GJB2, GJB6 and SLC26A4 genes in Korean deafness patients. Int J Pediatr Otorhinolaryngol.

[46] Usami S, Nishio SY, Nagano M, Abe S, Yamaguchi T, Deafness Gene Study C (2012). Simultaneous screening of multiple mutations by invader assay improves molecular diagnosis of hereditary hearing loss: a multicenter study. PLoS One.

[47] Morton NE (1991). Genetic epidemiology of hearing impairment. Ann N Y Acad Sci.

[48] Shearer AE, Black-Ziegelbein EA, Hildebrand MS, Eppsteiner RW, Ravi H, Joshi S, Guiffre AC, Sloan CM, Happe S, Howard SD, Novak B, Deluca AP, Taylor KR, Scheetz TE, Braun TA, Casavant TL, Kimberling WJ, Leproust EM, Smith RJ (2013). Advancing genetic testing for deafness with genomic technology. J Med Genet.

[49] Rehman AU, Santos-Cortez RL, Morell RJ, Drummond MC, Ito T, Lee K, Khan AA, Basra MA, Wasif N, Ayub M, Ali RA, Raza SI, Nickerson DA, Shendure J, Bamshad M, Riazuddin S, Billington N, Khan SN, Friedman PL, Griffith AJ, Ahmad W, Riazuddin S, Leal SM, Friedman TB, University of Washington Center for Mendelian Genomics (2014). Mutations in TBC1D24, a gene associated with epilepsy, also cause nonsyndromic deafness DFNB86. Am J Hum Genet.

[50] Tekin M, Chioza BA, Matsumoto Y, Diaz-Horta O, Cross HE, Duman D, Kokotas H, Moore-Barton HL, Sakoori K, Ota M, Odaka YS, Foster J, Cengiz FB, Tokgoz-Yilmaz S, Tekeli O, Grigoriadou M, Petersen MB, Sreekantan-Nair A, Gurtz K, Xia XJ, Pandya A, Patton MA, Young JI, Aruga J, Crosby AH (2013). SLITRK6 mutations cause myopia and deafness in humans and mice. J Clin Invest.

[51] Santos-Cortez RL, Lee K, Azeem Z, Antonellis PJ, Pollock LM, Khan S, Irfanullah, Andrade-Elizondo PB, Chiu I, Adams MD, Basit S, Smith JD, Nickerson DA, McDermott BM, Ahmad W, Leal SM, University of Washington Center for Mendelian Genomics (2013). Mutations in KARS, encoding lysyl-tRNA synthetase, cause autosomal-recessive nonsyndromic hearing impairment DFNB89. Am J Hum Genet.

[52] Yariz KO, Duman D, Seco CZ, Dallman J, Huang M, Peters TA, Sirmaci A, Lu N, Schraders M, Skromne I, Oostrik J, Diaz-Horta O, Young JI, Tokgoz-Yilmaz S, Konukseven O, Shahin H, Hetterschijt L, Kanaan M, Oonk AM, Edwards YJ, Li H, Atalay S, Blanton S, Desmidt AA, Liu XZ, Pennings RJ, Lu Z, Chen ZY, Kremer H, Tekin M (2012). Mutations in OTOGL, encoding the inner ear protein otogelin-like, cause moderate sensorineural hearing loss. Am J Hum Genet.

[53] Delmaghani S, Aghaie A, Michalski N, Bonnet C, Weil D, Petit C (2012). Defect in the gene encoding the EAR/EPTP domain-containing protein TSPEAR causes DFNB98 profound deafness. Hum Mol Genet.

[54] Schraders M, Haas SA, Weegerink NJ, Oostrik J, Hu H, Hoefsloot LH, Kannan S, Huygen PL, Pennings RJ, Admiraal RJ, Kalscheuer VM, Kunst HP, Kremer H (2011). Next-generation sequencing identifies mutations of SMPX, which encodes the small muscle protein, X-linked, as a cause of progressive hearing impairment. Am J Hum Genet.

[55] Huebner AK, Gandia M, Frommolt P, Maak A, Wicklein EM, Thiele H, Altmuller J, Wagner F, Vinuela A, Aguirre LA, Moreno F, Maier H, Rau I, Giesselmann S, Nürnberg G, Gal A, Nürnberg P, Hübner CA, del Castillo I, Kurth I (2011). Nonsense mutations in SMPX, encoding a protein responsive to physical force, result in X-chromosomal hearing loss. Am J Hum Genet.

[56] Fowler KB, McCollister FP, Dahle AJ, Boppana S, Britt WJ, Pass RF (1997). Progressive and fluctuating sensorineural hearing loss in children with asymptomatic congenital cytomegalovirus infection. J Pediatr.

[57] Choi BY, An YH, Park JH, Jang JH, Chung HC, Kim AR, Lee JH, Kim CS, Oh SH, Chang SO (2013). Audiological and surgical evidence for the presence of a third window effect for the conductive hearing loss in DFNX2 deafness irrespective of types of mutations. Eur Arch Otorhinolaryngol.

[58] Choi JW, Min B, Kim AR, Koo JW, Kim CS, Park WY, Chung J, Kim V, Ryu YJ, Kim SH, Chang SO, Oh SH, Choi BY: **De novo large genomic deletions involving POU3F4 in incomplete partition type III inner Ear anomaly in east Asian populations and implications for genetic counseling.** *Otology & Neurotology* 2014, 10.1097/MAO.0000000000000343 24608376

[59] Choi BY, Kim DH, Chung T, Chang M, Kim EH, Kim AR, Seok J, Chang SO, Bok J, Kim D, Oh SH, Park WY (2013). Destabilization and mislocalization of POU3F4 by C-terminal frameshift truncation and extension mutation. Hum Mutat.

[60] Splawski I, Shen J, Timothy KW, Lehmann MH, Priori S, Robinson JL, Moss AJ, Schwartz PJ, Towbin JA, Vincent GM, Keating MT (2000). Spectrum of mutations in long-QT syndrome genes. KVLQT1, HERG, SCN5A, KCNE1, and KCNE2. Circulation.

