# Supplementary material for: Exploration of molecular genetic etiology for Korean cochlear implantees with severe to profound hearing loss and its implication
Source: Orphanet J Rare Dis. 2014 Nov 6;9:167. doi: 10.1186/s13023-014-0167-8 (PMC4243193; doi:10.1186/s13023-014-0167-8)
Supplement: Additional file 1: Table S1. — 204 deafness genes. Table S2. Details of variants in SLC26A4, POU3F4, OTOF and KCNQ1 detected from the phenotype driven candidate gene approach. Table S3. Summary statistics of targeted exome sequencing for 45 samples [57–60]. [file 13023_2014_167_MOESM1_ESM.docx]

**Table S1. 204 deafness genes**

| **No** | **Gene** | **mRNA** | **CDS** | **Locus** | **Evidence** |  | **No** | **Gene** | **mRNA** | **CDS** | **Locus** | **Evidence** |  | **No** | **Gene** | **mRNA** | **CDS** | **Locus** | **Evidence** |
| --- | --- | --- | --- | --- | --- | --- | --- | --- | --- | --- | --- | --- | --- | --- | --- | --- | --- | --- | --- |
| 1 | ACTB | 1852 | 1128 |  | NCBI |  | 69 | GJB6 | 1805 | 785 |  | OMIM |  | 137 | PDZD7 | 2072 | 1554 |  | NCBI |
| 2 | ACTG1 | 1919 | 1127 |  | OMIM |  | 70 | GLI2 |  |  |  | expression |  | 138 | PEJVAKIN | 1415 | 1058 |  | NCBI |
| 3 | ALDH1A2 |  |  | 15q21.3 | expression |  | 71 | GLI3 |  |  |  | expression |  | 139 | PHEX |  |  |  | expression |
| 4 | APAF1 |  |  |  | expression |  | 72 | GPR98 |  |  |  | expression |  | 140 | PLAT |  |  | 8p12 | OMIM |
| 5 | ATF2 |  |  |  | expression |  | 73 | GRHL2 | 5231 | 1878 |  | NCBI |  | 141 | PLDN |  |  | 15q15 | expression |
| 6 | ATOH1 |  |  |  | expression |  | 74 | GRID1 |  |  |  | expression |  | 142 | PMP22 | 1828 | 483 |  | NCBI |
| 7 | ATP2B2 |  |  |  | expression |  | 75 | GSC |  |  |  | expression |  | 143 | PON1 |  |  | 7q21.3 | OMIM |
| 8 | ATP6V1B1 | 1956 | 1542 |  | NCBI |  | 76 | GSTP1 | 986 | 633 |  | NCBI |  | 144 | PON2 |  |  | 7q21.3 | OMIM |
| 9 | AXIN1 |  |  | 16p13.3 | expression |  | 77 | HAPLN1 |  |  | 5q13-q14.1 | expression |  | 145 | POU1F1 |  |  |  | expression |
| 10 | BCS1L | 1663 | 1230 |  | NCBI |  | 78 | HMX2 |  |  |  | expression |  | 146 | POU3F4 | 1491 | 1086 |  | OMIM |
| 11 | BDNF |  |  |  | expression |  | 79 | HMX3 |  |  |  | expression |  | 147 | POU4F1 |  |  |  | expression |
| 12 | BMP4 |  |  |  | expression |  | 80 | HOXA1 |  |  |  | expression |  | 148 | POU4F3 | 1017 | 1017 |  | NCBI |
| 13 | BMP5 |  |  |  | expression |  | 81 | HOXA2 |  |  |  | expression |  | 149 | PRODH |  |  |  | expression |
| 14 | BSND | 1396 | 963 |  | OMIM |  | 82 | HOXB1 |  |  |  | expression |  | 150 | PROP1 |  |  |  | expression |
| 15 | CACNG2 |  |  |  | expression |  | 83 | HOXB2 |  |  |  | expression |  | 151 | PRRX1 |  |  | 1q24 | expression |
| 16 | CAT |  |  | 11p13 | OMIM |  | 84 | HSPG2 |  |  |  | expression |  | 152 | PRRX2 |  |  | 9q34.1 | expression |
| 17 | CATSPER2 | 1948 | 1593 |  | NCBI |  | 85 | ITGA1 |  |  | 5q11.2 | OMIM |  | 153 | RARA |  |  |  | expression |
| 18 | CCDC50 | 2454 | 1449 |  | NCBI |  | 86 | ITGA3 |  |  | 17q21.33 | OMIM |  | 154 | RARB |  |  |  | expression |
| 19 | CDH23 | 11073 | 10065 |  | NCBI |  | 87 | ITGA8 |  |  |  | expression |  | 155 | RARG |  |  | 12q13 | expression |
| 20 | CDKN1B |  |  |  | expression |  | 88 | JAG1 | 5988 | 3657 |  | NCBI |  | 156 | RDX | 4498 | 1752 |  | NCBI |
| 21 | CLDN14 | 1943 | 720 |  | NCBI |  | 89 | JAG2 |  |  |  | expression |  | 157 | RELN |  |  |  | expression |
| 22 | CLRN1 |  |  | 3q21-q25 | expression |  | 90 | KCNE1 | 3338 | 390 |  | NCBI |  | 158 | RNR1 |  |  | 13p12 | OMIM |
| 23 | COCH | 2534 | 1652 |  | NCBI |  | 91 | KCNJ10 | 5323 | 1140 |  | NCBI |  | 159 | RORA |  |  |  | expression |
| 24 | COL11A1 |  |  |  | expression |  | 92 | KCNQ4 | 2335 | 2087 |  | NCBI |  | 160 | SERPINE1 |  |  | 7q21.3-q22 | OMIM |
| 25 | COL11A2 | 6414 | 5210 |  | NCBI |  | 93 | KIT |  |  |  | expression |  | 161 | SLC12A2 |  |  |  | expression |
| 26 | COL1A1 |  |  | 17q21.33 | OMIM |  | 94 | KRML |  |  | 20q11.2-q13.1 | expression |  | 162 | SLC17A8 | 3983 | 1770 |  | NCBI |
| 27 | COL1A2 |  |  | 7q22.1 | expression |  | 95 | LAMA2 |  |  |  | expression |  | 163 | SLC26A4 | 4930 | 2343 |  | OMIM |
| 28 | COL2A1 |  |  |  | expression |  | 96 | LHFPL5 | 2162 | 659 |  | NCBI |  | 164 | SLC26A5 | 2671 | 2235 |  | NCBI |
| 29 | COL4A3 |  |  |  | expression |  | 97 | LHX3 | 2376 | 1194 |  | NCBI |  | 165 | SLC30A4 |  |  |  | expression |
| 30 | COL9A3 | 2485 | 2055 |  | NCBI |  | 98 | LMX1A |  |  |  | expression |  | 166 | SLC4A11 | 3110 | 2379 |  | NCBI |
| 31 | CRYM | 1303 | 945 |  | NCBI |  | 99 | LRTOMT | 2332 | 579 |  | NCBI |  | 167 | SLC9A1 |  |  |  | expression |
| 32 | CSF1 |  |  |  | expression |  | 100 | MARVELD2 |  |  | 5q13.1 | expression |  | 168 | SNAP25 |  |  |  | expression |
| 33 | DFNA5 | 2230 | 1490 |  | NCBI |  | 101 | MBP |  |  |  | expression |  | 169 | SOD1 |  |  |  | expression |
| 34 | DFNB31 | 4022 | 2723 |  | NCBI |  | 102 | MITF |  |  |  | expression |  | 170 | SOD2 |  |  | 6q25.3 | OMIM |
| 35 | DFNB59 |  |  | 2q31.1-q31.3 | expression |  | 103 | MOS |  |  |  | expression |  | 171 | SOX10 |  |  |  | NCBI |
| 36 | DIAPH1 | 5662 | 3746 |  | NCBI |  | 104 | MPV17 |  |  |  | expression |  | 172 | SOX2 | 2518 | 924 |  | NCBI |
| 37 | DLX1 |  |  |  | expression |  | 105 | MPZ |  |  |  | expression |  | 173 | SPINK5 | 3655 | 3195 |  | NCBI |
| 38 | DLX2 |  |  |  | expression |  | 106 | MSX1 |  |  |  | expression |  | 174 | STRC | 5516 | 5328 |  | NCBI |
| 39 | DLX5 |  |  |  | expression |  | 107 | MSX2 |  |  |  | expression |  | 175 | TBL1X | 5586 | 1581 |  | NCBI |
| 40 | DMD |  |  |  | expression |  | 108 | MTAP | 4937 | 852 |  | NCBI |  | 176 | TCF21 | 3249 | 540 |  | NCBI |
| 41 | DSPP | 4187 | 3761 |  | NCBI |  | 109 | MTHFR |  |  | 1p36.3 | OMIM |  | 177 | TECTA | 6469 | 6468 |  | NCBI |
| 42 | ECE1 |  |  |  | expression |  | 110 | MT-RNR1 |  |  | mitochondria | OMIM |  | 178 | TFAP2A |  |  | 6p24 | expression |
| 43 | EDN1 |  |  |  | expression |  | 111 | MYH14 | 6786 | 5987 |  | NCBI |  | 179 | TFCP2L3 | 4793 | 1877 |  | NCBI |
| 44 | EDN3 |  |  |  | expression |  | 112 | MYH9 | 7474 | 5882 |  | OMIM |  | 180 | TGFA |  |  |  | expression |
| 45 | EDNRA |  |  |  | expression |  | 113 | MYO15A | 11876 | 10593 |  | NCBI |  | 181 | TGFB2 |  |  |  | expression |
| 46 | EDNRB |  |  |  | expression |  | 114 | MYO1A | 3624 | 3131 |  | NCBI |  | 182 | THRA |  |  |  | expression |
| 47 | ERCC2 | 2568 | 2283 |  | NCBI |  | 115 | MYO1C | 4973 | 3192 |  | NCBI |  | 183 | THRB |  |  |  | expression |
| 48 | ERCC3 | 2751 | 2349 |  | NCBI |  | 116 | MYO1F | 4173 | 3297 |  | NCBI |  | 184 | TIMM8A | 1459 | 294 |  | NCBI |
| 49 | ESPN | 3542 | 2564 |  | OMIM |  | 117 | MYO3A | 5597 | 4850 |  | NCBI |  | 185 | TMC1 | 3201 | 2283 |  | NCBI |
| 50 | ESRRB | 2193 | 2193 |  | NCBI |  | 118 | MYO6 | 5278 | 3857 |  | OMIM |  | 186 | TMIE | 1646 | 468 |  | NCBI |
| 51 | EYA1 |  |  |  | expression |  | 119 | MYO7A | 7465 | 6648 |  | NCBI |  | 187 | TMPRSS3 | 2468 | 1365 |  | OMIM |
| 52 | EYA4 | 3077 | 1919 |  | NCBI |  | 120 | NDP |  |  | Xp11.4 | expression |  | 188 | TMPRSS5 | 2233 | 1374 |  | NCBI |
| 53 | F2 |  |  | 11p11 | OMIM |  | 121 | NEUROG1 |  |  | 5q23-q31 | expression |  | 189 | TNC |  |  |  | expression |
| 54 | F5 |  |  | 1q23 | OMIM |  | 122 | NF2 |  |  |  | expression |  | 190 | TRIC | 2385 | 1374 |  | NCBI |
| 55 | FAS |  |  |  | expression |  | 123 | NR2F1 | 3210 | 1272 |  | NCBI |  | 191 | TRIOBP | 10024 | 7097 |  | NCBI |
| 56 | FGF3 | 1548 | 720 |  | NCBI |  | 124 | NTF3 |  |  |  | expression |  | 192 | TRMU |  |  | 22q13 | NCBI |
| 57 | FGF8 |  |  |  | expression |  | 125 | NTN1 |  |  |  | expression |  | 193 | TRNL1 |  |  | mitochondria | OMIM |
| 58 | FGFR2 |  |  |  | expression |  | 126 | OTOA | 3625 | 3420 |  | NCBI |  | 194 | TRNS1 |  |  | mitochondria | OMIM |
| 59 | FGFR3 |  |  |  | expression |  | 127 | OTOF | 7173 | 5994 |  | NCBI |  | 195 | TSHR |  |  |  | expression |
| 60 | FOXI1 |  |  | 5q34 | expression |  | 128 | OTOR | 1477 | 387 |  | NCBI |  | 196 | TUB |  |  |  | expression |
| 61 | GATA3 | 3067 | 1002 |  | NCBI |  | 129 | OTX1 |  |  |  | expression |  | 197 | TYMP |  |  | 22q13.33 | OMIM |
| 62 | GJA1 | 3130 | 1149 |  | NCBI |  | 130 | OTX2 |  |  |  | expression |  | 198 | TYR |  |  |  | expression |
| 63 | GJA4 |  |  | 1p35.1 | NCBI |  | 131 | P73 |  |  | 1q36.3 | expression |  | 199 | TYRP1 |  |  |  | expression |
| 64 | GJB1 | 1623 | 852 |  | NCBI |  | 132 | PAX2 |  |  |  | expression |  | 200 | UCN |  |  | 2p23-p21 | OMIM |
| 65 | GJB2 | 2263 | 681 |  | OMIM |  | 133 | PAX3 | 3359 | 1455 |  | NCBI |  | 201 | USH1C | 2228 | 2700 |  | NCBI |
| 66 | GJB3 | 1777 | 820 |  | OMIM |  | 134 | PAX5 |  |  |  | expression |  | 202 | USH1G |  |  | 17q24-q25 | expression |
| 67 | GJB4 | 2840 | 801 |  | NCBI |  | 135 | PAX9 |  |  |  | expression |  | 203 | USH2A |  |  |  | OMIM |
| 68 | GJB5 |  |  | 1p35.1 | NCBI |  | 136 | PCDH15 | 7022 | 5868 |  | NCBI |  | 204 | WFS1 | 3640 | 2670 |  | NCBI |

NCBI, National Center for Biotechnology Information; OMIM, Online Mendelian Inheretance in Man **Table S2.**Details of variants in *SLC26A4*, *POU3F4*, *OTOF* and *KCNQ1* detected from the phenotype driven candidate gene approach

| **Patient** | **Characteristic Phenotype** | **Gene** | **Mutation type** | **Gene Bank No.** | **Chr** | **Exon** | **Nucleotide** | **Protein** | **dbSNP137/Reference** |
| --- | --- | --- | --- | --- | --- | --- | --- | --- | --- |
| **SHJ7** | Enlarged Vestibular aqueduct | SLC26A4 | nonsynonymous SNV | NM_000441 | 7 | 19 | c.A2168G | p.H723R  (homo) | rs121908362 |
| **SHJ8** | Enlarged Vestibular aqueduct | SLC26A4 | nonsynonymous SNV | NM_000441 | 7 | 19 | c.A2168G | p.H723R  (homo) | rs121908362 |
| **SHJ11** | Enlarged Vestibular aqueduct | SLC26A4 | Splicing | NM_000441 | 7 | Intron7 | c.919-2A>G | - | rs111033313 |
|  |  | SLC26A4 | nonsynonymous SNV | NM_000441 | 7 | 19 | c.A2168G | p.H723R | rs121908362 |
| **SHJ17** | Enlarged Vestibular aqueduct | SLC26A4 | Splicing | NM_000441 | 7 | Intron7 | c.919-2A>G | - | rs111033313 |
|  |  |  | nonsynonymous SNV | NM_000441 | 7 | 13 | c.G1489A | p.G497S | rs111033308 |
| **SHJ32** | Enlarged Vestibular aqueduct | SLC26A4 | Splicing | NM_000441 | 7 | Intron7 | c.919-2A>G | - | rs111033313 |
|  |  |  | nonsynonymous SNV | NM_000441 | 7 | 19 | c.A2168G | p.H723R | rs121908362 |
| **SNUH18-44** | Enlarged Vestibular aqueduct | SLC26A4 | nonsynonymous SNV | NM_000441 | 7 | 16 | c.1714T>C | p.F572L | This study |
|  |  |  | nonsynonymous SNV | NM_000441 | 7 | 19 | c.A2168G | p.H723R | rs121908362 |
| SNUH36-77 (SHJ20) | Enlarged Vestibular aqueduct | SLC26A4 | nonsynonymous SNV | NM_000441 | 7 | 19 | c.A2168G | p.H723R  (homo) | rs121908362 |
| SNUBH12-28 | Enlarged Vestibular aqueduct | SLC26A4 | Splicing | NM_000441 | 7 | Intron7 | c.919-2A>G | - | rs111033313 |
|  |  |  | nonsynonymous | NM_000441 | 7 | 19 | c.A2168G | p.H723R | rs121908362 |
| SNUBH17-38 | Enlarged Vestibular aqueduct | SLC26A4 | nonsynonymous SNV | NM_000441 | 7 | 19 | c.A2168G | p.H723R  (homo) | rs121908362 |
| SNUBH20-47 | Enlarged Vestibular aqueduct | SLC26A4 | nonsynonymous SNV | NM_000441 | 7 | 19 | c.A2168G | p.H723R  (homo) | rs121908362 |
| SNUBH23-54 | Enlarged Vestibular aqueduct | SLC26A4 | nonsynonymous SNV | NM_000441 | 7 | 10 | c.1229C>T | p.T410M | rs111033220 |
|  |  |  | nonsynonymous SNV | NM_000441 | 7 | 19 | c.A2168G | p.H723R | rs121908362 |
| SNUBH23-55 | Enlarged Vestibular aqueduct | SLC26A4 | Splicing | NM_000441 | 7 | Intron7 | c.919-2A>G | - | rs111033313 |
|  |  |  | nonsynonymous SNV | NM_000441 | 7 | 10 | c.1229C>T | p.T410M | rs111033220 |
| SNUBH58-105 | Enlarged Vestibular aqueduct | SLC26A4 | nonsynonymous SNV | NM_000441 | 7 | 19 | c.A2168G | p.H723R  (homo) | rs121908362 |
| SNUH17-42 | Incomplete partition type III | POU3F4 | Large genomic deletion | NM_000307 | X | - | - | - | [57] |
| SNUH65-151 | Incomplete partition type III | POU3F4 | nonsynonymous SNV | NM_000307 | X | 1 | c.908C>A | p.P303H | [58] |
| SNUBH2-4 | Incomplete partition type III | POU3F4 | nonsynonymous SNV | NM_000307 | X | 1 | c.686A>G | p.Q229R | [59] |
| SNUBH13-29 | Incomplete partition type III | POU3F4 | nonsynonymous SNV | NM_000307 | X | 1 | c.623T>A | p.L208X | [59] |
| SNUBH19-44 | Incomplete partition type III | POU3F4 | Large genomic deletion | NM_000307 | X | - | - | - | [58] |
| SNUBH4-12 | Auditory neuropathy/Auditory dyssynchrony | OTOF | stopgain | NM_194322 | 2 | 8 | c.C1122G | p.Y374X | [20] |
|  |  |  | nonsynonymous SNV | NM_194322 | 2 | 24 | c.3133delC | p.R1045Gfs*28 | [20] |
| SNUH78-179 (SHJ57) | Jervell Lange  Nielson | KCNQ1 | duplication | NM_000218 | 11 | 16 | c.1893dupC | p.Arg632Glnfs*20 | [60] |
|  |  |  | deletion | NM_000218 | 11 | 7-10 | * | * | This study |

* The large deletion is encompassed from exon7 to exon10 in KCNQ1 gene.

**Table S3.** Summary statistics of targeted exome sequencing for 45 samples

1. **CI implantees with an unknown etiology after molecular genetic test (n=34)**

| **SAMPLE:** | **SNUH41-90** | **SHJ1** | **SHJ3** | **SHJ6** | **SHJ9** | **SHJ10** | **SHJ13** | **SHJ14** | **SHJ15** |
| --- | --- | --- | --- | --- | --- | --- | --- | --- | --- |
| TOTAL READS: | 15110682 | 8145256 | 11080656 | 6215070 | 4121704 | 5162368 | 3099494 | 3806896 | 17052 |
| UNIQUE READS ALIGNED: | 10955279 | 6746576 | 7883015 | 3324116 | 2074155 | 2650096 | 2495504 | 3021105 | 13503 |
| ON TARGET BASES: | 414173721 | 258961887 | 279494144 | 120170848 | 81901360 | 100717938 | 96918512 | 113437101 | 520462 |
| TARGET COVERAGE: | 501.782498 | 314.553441 | 339.265598 | 145.943243 | 99.554445 | 122.26404 | 117.816102 | 137.726298 | 79.1954 |
| ZERO COVERAGE TARGETS %: | 0.005283 | 0.007044 | 0.00587 | 0.007044 | 0.007925 | 0.006751 | 0.008218 | 0.006457 | 0.915762 |
| TARGET BASES 20X %: | 0.964875 | 0.956193 | 0.952962 | 0.940833 | 0.931994 | 0.943879 | 0.941941 | 0.947912 | 0.006147 |
| TARGET BASES 50X %: | 0.938217 | 0.913343 | 0.906712 | 0.856587 | 0.817665 | 0.850457 | 0.850898 | 0.872415 | 0.005512 |
| TARGET BASES 100X %: | 0.885991 | 0.831734 | 0.825258 | 0.671836 | 0.504233 | 0.610843 | 0.60611 | 0.686891 | 0.002845 |
|  |  |  |  |  |  |  |  |  |  |
| **SAMPLE:** | **SHJ16** | **SNUH38-86** | **SHJ22** | **SHJ25** | **SHJ27** | **SHJ28** | **SHJ29** | **SHJ35** | **SHJ55** |
| TOTAL READS: | 2405058 | 12619174 | 3668926 | 3577924 | 2695165 | 2704755 | 2974920 | 3556361 | 4067301 |
| UNIQUE READS ALIGNED: | 2049355 | 9975001 | 3592236 | 3507503 | 2642607 | 2647427 | 2907343 | 3476065 | 3979326 |
| ON TARGET BASES: | 76546195 | 380168143 | 205582961 | 200598194 | 152275965 | 150114437 | 162865099 | 198907121 | 224376910 |
| TARGET COVERAGE: | 93.112133 | 460.883025 | 249.589728 | 243.549707 | 185.011913 | 182.553364 | 198.135599 | 241.490257 | 272.498872 |
| ZERO COVERAGE TARGETS %: | 0.008218 | 0.004696 | 0.006457 | 0.006457 | 0.007338 | 0.008805 | 0.008218 | 0.006457 | 0.007631 |
| TARGET BASES 20X %: | 0.925131 | 0.964605 | 0.952952 | 0.951902 | 0.939932 | 0.942053 | 0.948139 | 0.954061 | 0.955887 |
| TARGET BASES 50X %: | 0.790061 | 0.940193 | 0.900688 | 0.893704 | 0.860251 | 0.863975 | 0.883751 | 0.902623 | 0.906788 |
| TARGET BASES 100X %: | 0.445995 | 0.890991 | 0.794721 | 0.785694 | 0.72243 | 0.727177 | 0.753949 | 0.803437 | 0.812825 |

1. **CI implantees with an unknown etiology after molecular genetic test (n=34) (Continued)**

| **SAMPLE:** | **SHJ63** | **SHJ64** | **SHJ69** | **SNUBH72_125** | **SNUH26_58** | **SNUH61_144** | **SNUH63_148** | **SNUH66_155** | **SNUH69_158** |
| --- | --- | --- | --- | --- | --- | --- | --- | --- | --- |
| TOTAL READS: | 3614598 | 3905292 | 3620169 | 3906340 | 3266257 | 3829791 | 3356713 | 3561254 | 3262611 |
| UNIQUE READS ALIGNED: | 3541307 | 3822903 | 3545116 | 3820593 | 3194945 | 3741968 | 3283970 | 3487230 | 3192888 |
| ON TARGET BASES: | 202190180 | 220816109 | 199894215 | 215528228 | 0.978167 | 0.977068 | 0.978329 | 0.979214 | 0.97863 |
| TARGET COVERAGE: | 245.466659 | 268.358413 | 242.73432 | 261.77404 | 179167747 | 210042579 | 186703117 | 201698628 | 1.86E+08 |
| ZERO COVERAGE TARGETS %: | 0.00587 | 0.007631 | 0.007044 | 0.007338 | 217.639017 | 255.05484 | 226.747648 | 244.810289 | 226.337 |
| TARGET BASES 20X %: | 0.952119 | 0.95403 | 0.952961 | 0.954071 | 0.007044 | 0.007044 | 0.007044 | 0.006164 | 0.006751 |
| TARGET BASES 50X %: | 0.897393 | 0.905538 | 0.897754 | 0.903204 | 0.95008 | 0.954459 | 0.951224 | 0.94862 | 0.94971 |
| TARGET BASES 100X %: | 0.7917 | 0.811743 | 0.794008 | 0.802283 | 0.886271 | 0.901777 | 0.897073 | 0.889816 | 0.889066 |
|  |  |  |  |  |  |  |  |  |  |
| **SAMPLE:** | **SNUH77_178** | **SNUH80_183** | **SNUH81_185** | **SNUH86_194** | **SNUH88_199** | **SNUH89_200** | **SNUBH22-51** |  |  |
| TOTAL READS: | 3523195 | 3256782 | 3973526 | 3533860 | 3486783 | 3277312 | 5696242 |  |  |
| UNIQUE READS ALIGNED: | 3448824 | 3187279 | 3884165 | 3454308 | 3407620 | 3205101 | 5010762 |  |  |
| ON TARGET BASES: | 196355800 | 180709498 | 222684023 | 199601421 | 195123705 | 186213274 | 51287955 |  |  |
| TARGET COVERAGE: | 238.419692 | 219.524426 | 270.258739 | 242.386202 | 236.798975 | 226.05725 | 167.463344 |  |  |
| ZERO COVERAGE TARGETS %: | 0.006457 | 0.006751 | 0.00587 | 0.006164 | 0.00587 | 0.006751 | 0.00403 |  |  |
| TARGET BASES 20X %: | 0.951478 | 0.950297 | 0.955828 | 0.951246 | 0.952482 | 0.948625 | 0.958445 |  |  |
| TARGET BASES 50X %: | 0.896049 | 0.890435 | 0.909263 | 0.89561 | 0.895933 | 0.890989 | 0.872442 |  |  |
| TARGET BASES 100X %: | 0.791301 | 0.773003 | 0.816391 | 0.788315 | 0.790794 | 0.777555 | 0.677478 |  |  |

1. **CI implantees with a definitive genetic diagnosis (n=11)**

| **SAMPLE:** | **SHJ4** | **SNUH3-7** | **SNUH10-28** | **SHJ23** | **SHJ33** | **SHJ37** | **SHJ38** | **SHJ70** | **SNUBH71_123** | **SNUH62_147** | **SNUH72_164** |
| --- | --- | --- | --- | --- | --- | --- | --- | --- | --- | --- | --- |
| TOTAL READS: | 3488312 | 948542 | 1385186 | 4372262 | 3691354 | 3177271 | 3524795 | 3275580 | 3918941 | 3994131 | 3102302 |
| UNIQUE READS ALIGNED: | 3032237 | 882261 | 1284476 | 4280404 | 3612923 | 3101283 | 3446274 | 3202931 | 3832292 | 3911435 | 3035481 |
| ON TARGET BASES: | 110166107 | 30123273 | 49436738 | 245638962 | 206802547 | 171880056 | 196199971 | 181772860 | 206897019 | 220615371 | 175170214 |
| TARGET COVERAGE: | 134.106379 | 36.885404 | 60.270917 | 298.157267 | 251.158444 | 208.752271 | 238.262169 | 220.775456 | 251.182287 | 268.129303 | 212.6629 |
| ZERO COVERAGE TARGETS %: | 0.009392 | 0.015556 | 0.01086 | 0.006457 | 0.006751 | 0.007044 | 0.006751 | 0.007044 | 0.006164 | 0.007925 | 0.006164 |
| TARGET BASES 20X %: | 0.909441 | 0.740467 | 0.832215 | 0.958013 | 0.952475 | 0.943362 | 0.952868 | 0.951615 | 0.94574 | 0.954832 | 0.946647 |
| TARGET BASES 50X %: | 0.789241 | 0.288595 | 0.589626 | 0.913762 | 0.896339 | 0.873008 | 0.895852 | 0.891085 | 0.880731 | 0.906391 | 0.882208 |
| TARGET BASES 100X %: | 0.617664 | 0.001046 | 0.150762 | 0.827958 | 0.791839 | 0.743924 | 0.787669 | 0.775187 | 0.771044 | 0.812549 | 0.760676 |
